# Supplementary material for: Widespread intronic polyadenylation diversifies immune cell transcriptomes
Source: Nat Commun. 2018 Apr 30;9:1716. doi: 10.1038/s41467-018-04112-z (PMC5928244; doi:10.1038/s41467-018-04112-z)
Supplement: Supplementary file 1 — Supplementary Information [file 41467_2018_4112_MOESM1_ESM.pdf]

Supplementary File

# Widespread intronic polyadenylation diversifies immune cell transcriptomes

Singh et al

Supplementary Figure 1

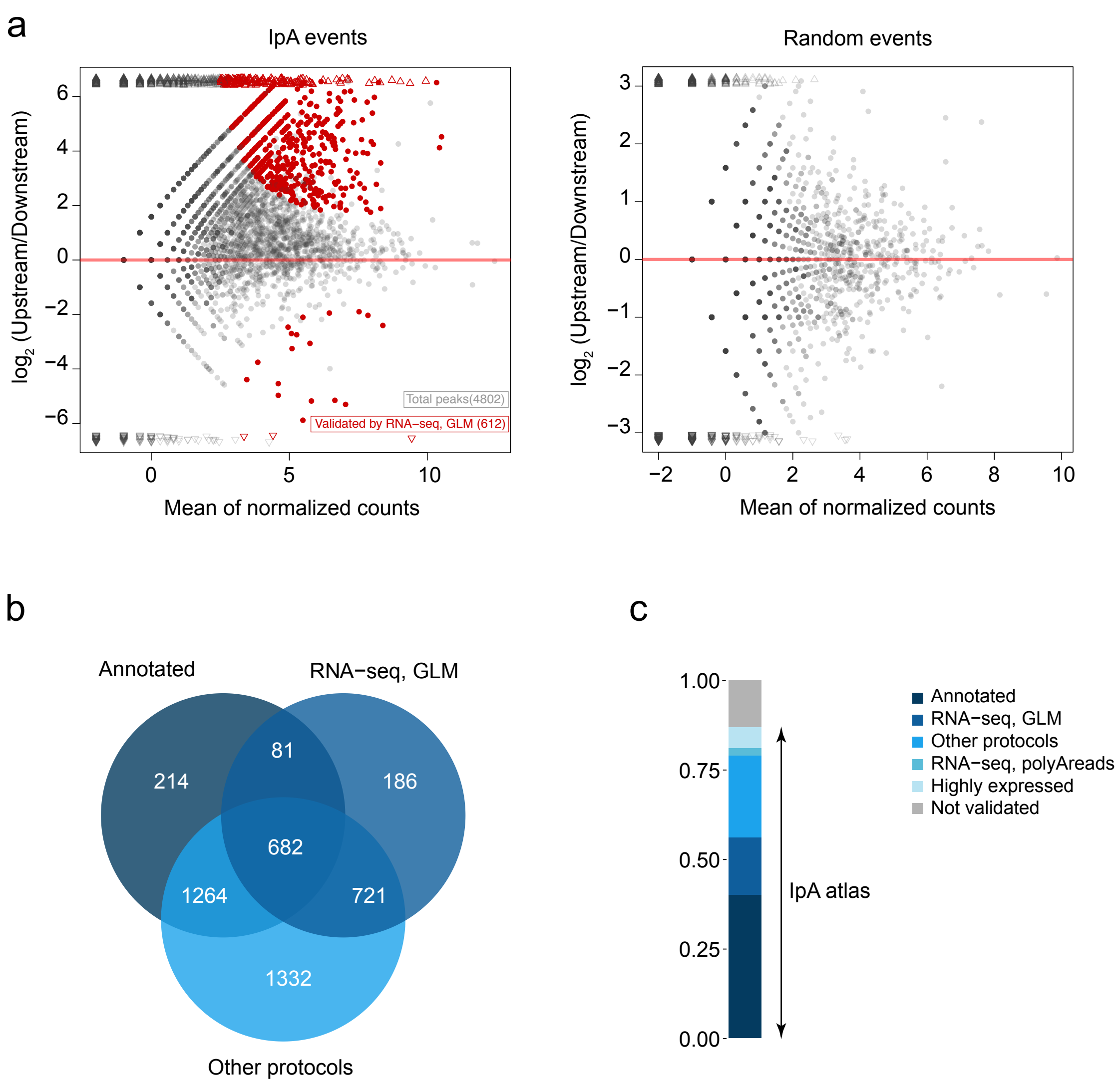

**Supplementary Figure 1: Validation of IpA events.**

a) Differential read coverage of RNA-seq data up- and downstream of IpA events was tested using a GLM. If significantly higher coverage was found upstream versus downstream of the IpA event, the IpA isoform was considered validated and highlighted in red (FDR-adjusted $P < 0.1$). The same test was performed on random events obtained up- and downstream of IpA sites without read evidence and did not yield significant events (right panel).

b) Venn diagram showing the number of events that were validated by RNA-seq, other 3' end sequencing protocols, and database annotation.

c) Atlas of high confidence IpA isoforms from 3'-seq. To validate IpA isoforms additional evidence from different sources was used sequentially. In the final IpA isoform atlas, 39.51% (n = 2,241) coincide with annotated isoforms in RefSeq, UCSC genes or Ensembl databases; 16.0% (n = 907) lack annotation but are validated by RNA-seq by the GLM-based test; 23.50% (n = 1,332) lack RNA-seq or annotation support but are reported in data sets generated with other 3' end sequencing protocols; 2.19% (n = 124) lack the previous sources of evidence but are corroborated by untemplated polyA reads using RNA-seq data; and the remaining 5.7% (n = 323) are highly expressed in at least one sample (> 10 TPM). 13.1% (n = 743) of IpA events could not be validated by any of these methods and were removed from further analyses.

# Supplementary Figure 2

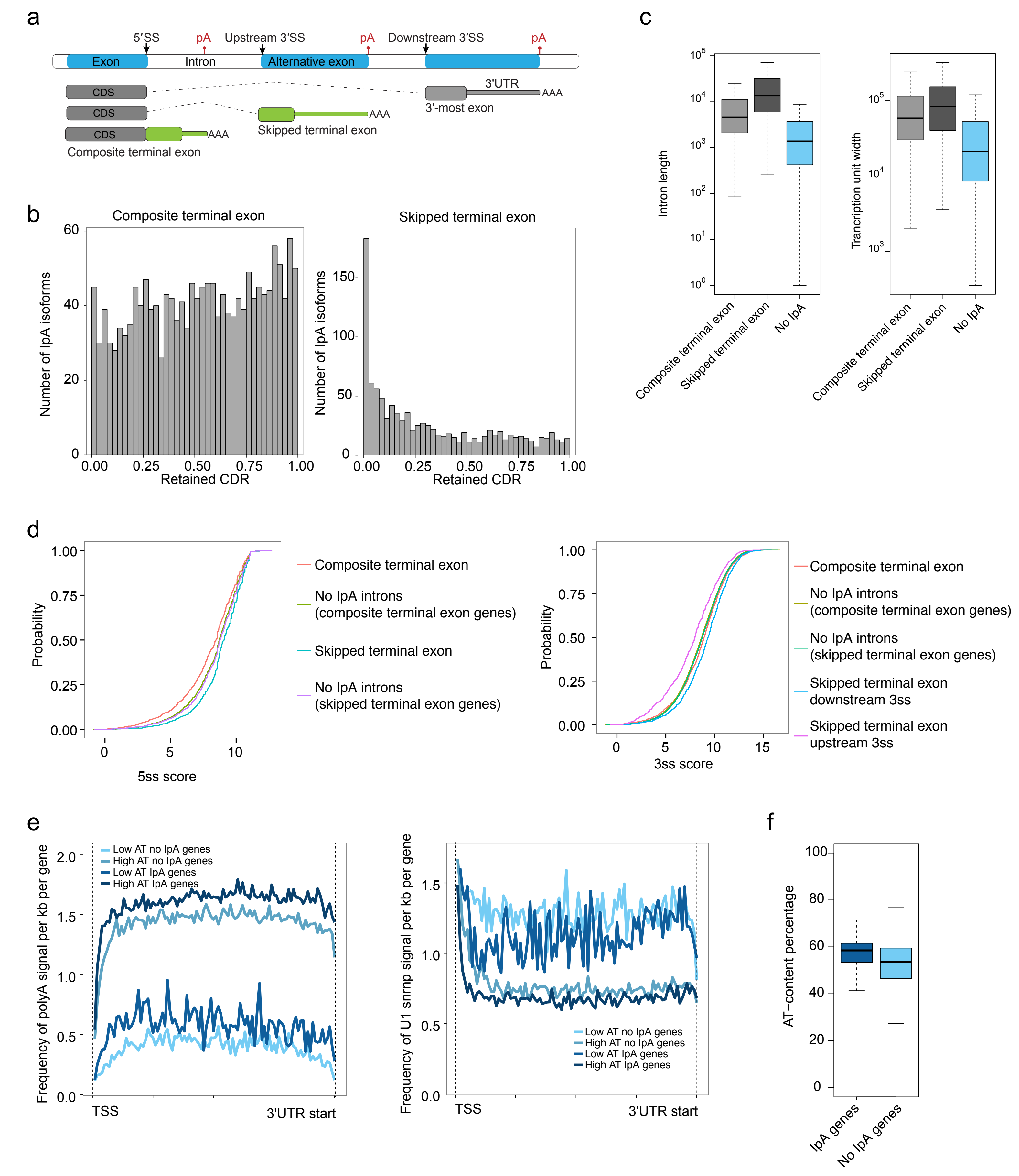

**Supplementary Figure 2: Composite vs skipped terminal IpA.**

a) Composite terminal exon events result from loss of recognition of 5'ss while skipped terminal exon results in inclusion of a new exon.

b) Composite terminal exon recognizes IpA sites uniformly across the transcription unit while skipped terminal exon are predominant near the TSS.

c) Skipped terminal exon occurs in longer introns compared to composite terminal exon or introns of genes with no IpA (one-sided Wilcoxon rank-sum test, $P < 10^{-20}$). They also occur in longer transcription units (one-sided Wilcoxon rank-sum test, $P < 10^{-20}$).

d) 5ss of composite terminal exon is weaker than the 5ss of the other introns of the same genes (one-sided KS test, $P < 10^{-09}$). 5ss of skipped terminal exon is stronger than the 5ss of the other introns of the same genes (one-sided KS test, $P < 10^{-06}$). Upstream 3ss of skipped terminal exon is weaker than the other introns of the same genes (one-sided KS test, $P < 10^{-15}$). Downstream 3ss of skipped terminal exon is weaker than the other introns of the same genes (one-sided KS test, $P < 10^{-09}$).

e) There is significant enrichment of pA signals in IpA genes vs. non-IpA genes in both high and low AT regions (one-sided Wilcoxon signed-rank test, IpA high-AT vs non-IpA_high-AT, $P < 10^{-17}$; IpA low-AT vs non-IpA_low-AT, $P < 10^{-16}$) as well as depletion of U1 signals (one-sided Wilcoxon signed-rank test, IpA high-AT vs non-IpA_high-AT, $P < 10^{-15}$; IpA low-AT vs non-IpA_low-AT, $P < 10^{-10}$)

f) IpA genes have higher AT-content compared to non-IpA genes (Wilcoxon rank-sum test, $P < 10^{-20}$).

# Supplementary Figure 3

A

Presence of domains in IpA vs no IpA genes

| | IpA genes | No IpA genes | Fisher-exact *p* |
|---|---|---|---|
| DNA binding present | 284 | 810 | 0.001 |
| DNA binding absent | 3147 | 11400 | |
| RNA binding present | 117 | 239 | $1.42 \times 10^{-6}$ |
| RNA binding absent | 3314 | 11971 | |
| PPI present | 794 | 2054 | 1.2E-16 |
| PPI absent | 2637 | 10156 | |
| TMD present | 673 | 2904 | $2.23 \times 10^{-7}$ |
| TMD absent | 2758 | 9306 | |
| Active site present | 435 | 1325 | 0.003 |
| Active site absent | 2996 | 10885 | |
| Repeats present | 994 | 2826 | $4.78 \times 10^{-12}$ |
| Repeats absent | 2437 | 9384 | |

B

Preferential loss of domains within IpA genes(n = 1,410)

| | | | Fisher-exact *p* |
|---|---|---|---|
| | DNA binding | Other domains | |
| Lost | 303 | 4859 | 0.0005 |
| Retained | 238 | 5194 | |
| | RNA binding | Other domains | |
| Lost | 46 | 5116 | 0.32 |
| Retained | 39 | 5393 | |
| | PPI | Other domains | |
| Lost | 931 | 4231 | $4.5 \times 10^{-7}$ |
| Retained | 783 | 4649 | |
| | TMD | Other domains | |
| Lost | 751 | 4411 | 0.10 |
| Retained | 852 | 4580 | |
| | Active site | Other domains | |
| Lost | 133 | 5029 | 0.04 |
| Retained | 175 | 5257 | |

C

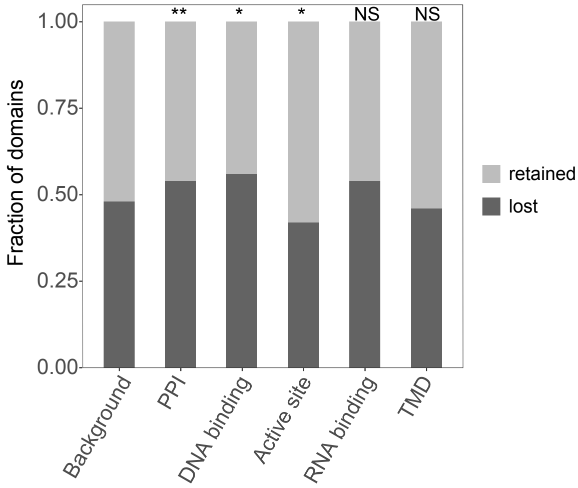

**Supplementary Figure 3: Statistics for domain enrichment in IpA genes.**

a) Domain information was obtained from UniProt and categorized (see Methods). IpA genes encode proteins enriched in DNA-binding, RNA-binding, PPI domains and active sites but are depleted for TMDs relative to genes that do not express IpA isoforms. Fisher's exact test was performed for enrichment *p* values.

b) Expression of IpA isoforms leads to preferential loss of DNA-binding and PPI domains but retains active sites of enzymes compared to the other domains present in IpA genes. Fisher's exact test was performed on IpA isoforms that retain at least one domain.

c) Fraction of domains lost and retained determined from (b)

# Supplementary Figure 4

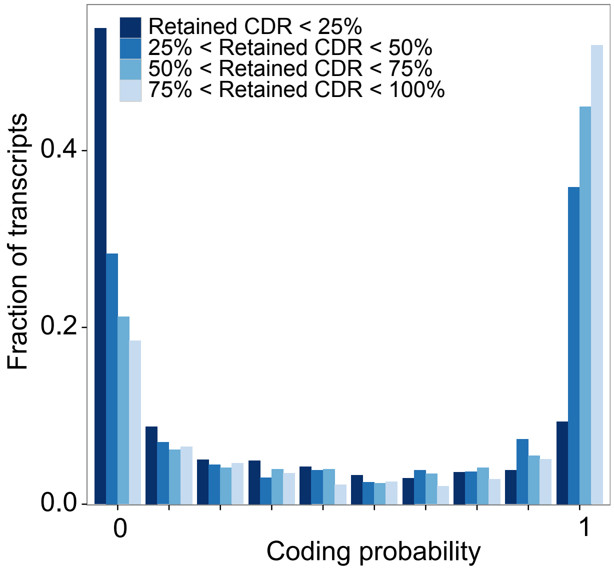

**Supplementary Figure 4: Predicted coding probability for IpA isoforms.**

Coding probability was assessed according to CPAT and shown for IpA isoforms with different fractions of retained CDR. Most of the IpA isoforms that retain less than 25% of the CDR are predicted to be non-coding.

Supplementary Figure 5

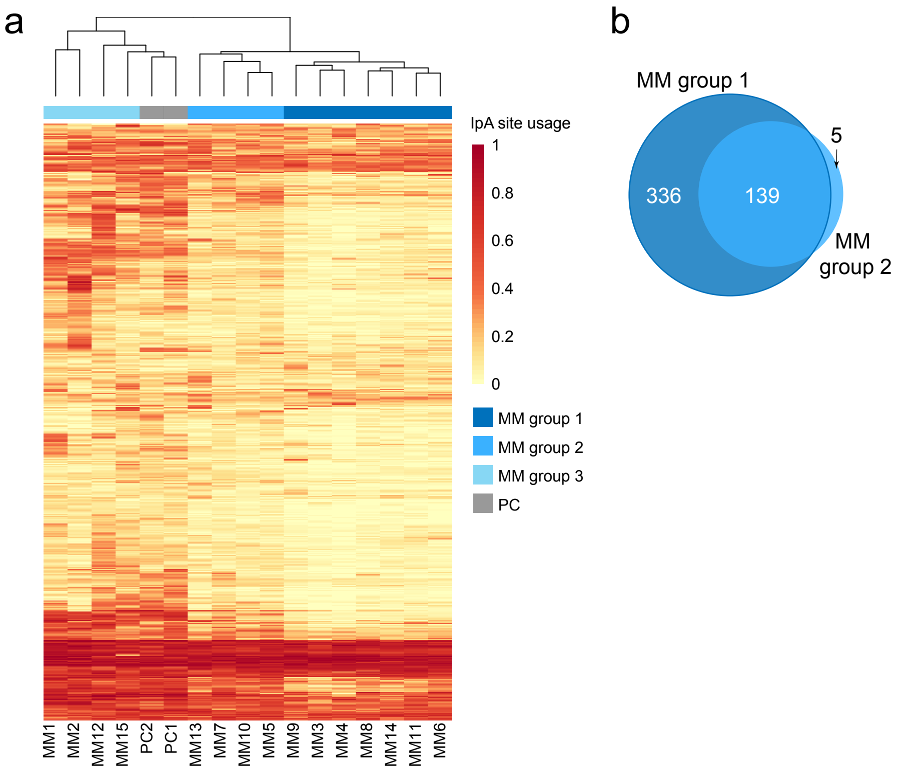

**Supplementary Figure 5: Hierarchical clustering defining MM patient groups.**

a) Hierarchical clustering of PCs and MM patient samples using 50% of the most variable IpA isoforms by median absolute deviation (n = 554) identifies three groups of MM samples.

b) Overlap of significantly lower used IpA sites in MM group 1 and group 2. MM group 3 is not shown as IpA site usage was very similar to PCs and only one IpA isoform was differentially used.

# Supplementary Figure 6

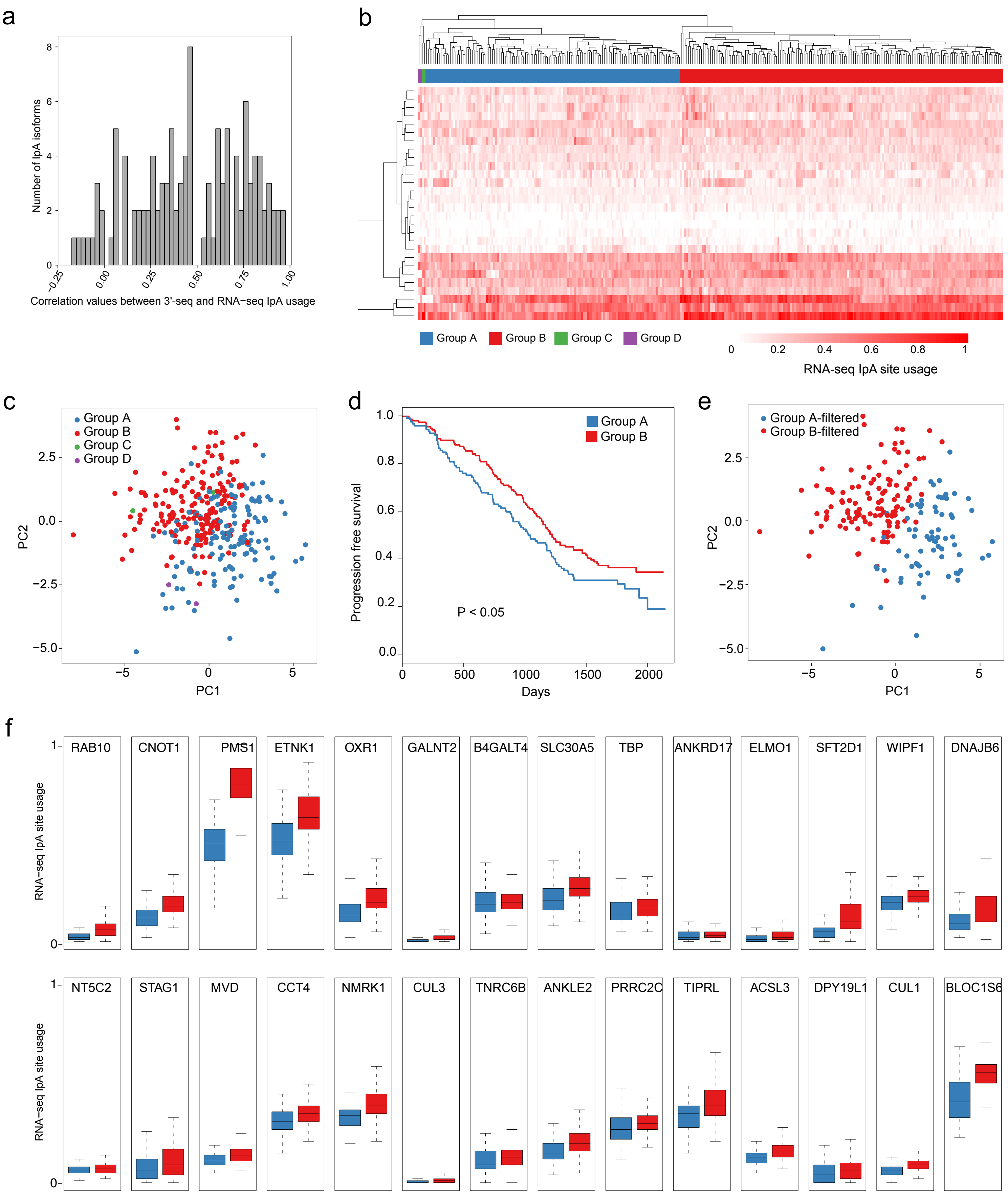

**Supplementary Figure 6: MM patients with high IpA usage have significantly improved progression free survival**

a) Distribution of correlation values of IpA usage determined from 3'-seq and RNA-seq for genes that do not recognize IpA sites in MM when compared to PCs (n = 114, FDR-adjusted $P < 0.05$, usage difference > 0.25)

b) Hierarchical clustering of RNA-seq IpA site usage of genes with high correlation between 3'-seq and RNA-seq usage (Pearson r > 0.75, n = 28) determined for an independent cohort of patients (n = 319) with RNA-seq expression. This clustering show separation of patients largely into two groups, group A – low IpA usage patients (n = 139) and group B - high IpA usage patients (n = 176).

c) Visualization of separation of patient groups by principal component analysis.

d) Group B patients with progression-free survival information (n = 160) have significantly improved ($P < 0.05$) progression free survival than group A patients (n = 126).

e) As (c) after removing heterogeneous samples from both groups, group A-filtered (n = 73) and group B-filtered (n = 115).

f) RNA-seq IpA site usage of the gene signature (n = 28) for samples of group A-filtered and group B-filtered from (e). Genes are ordered from high correlation between RNA-seq and 3'-seq IpA site usage.

Supplementary Figure 7

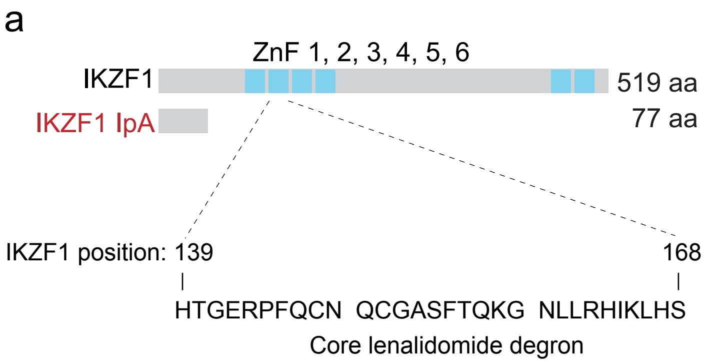


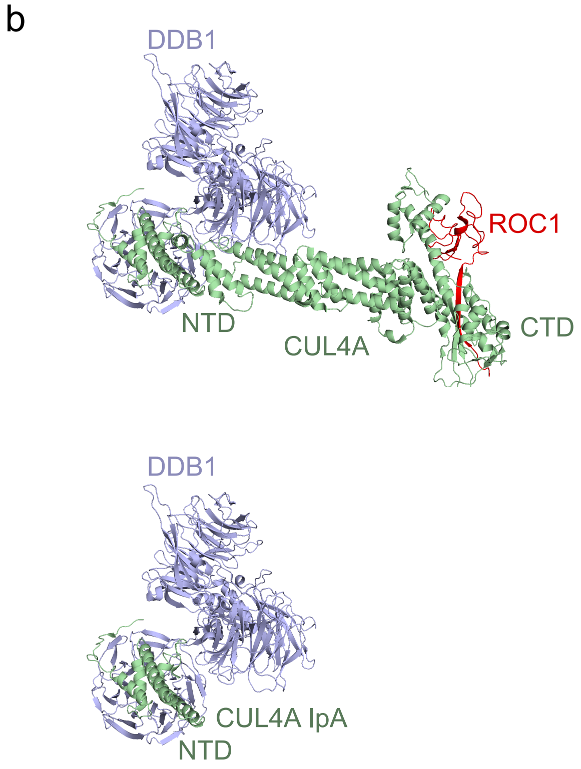

**Supplementary Figure 7: Potential role of IKZF1-IpA and CUL4A-IpA expression in resistance to lenalidomide**

b) Loss of core lenalidomide degron sequence from IKZF1 IpA.

a) DDB1-CUL4A-ROC1 complex (PDB id: 2HYE) and DDB1-CUL4A IpA complex. Full-length CUL4A has 41-759 aa (top) while CUL4A IpA has 41-171 aa (bottom).

# Supplementary Figure 8

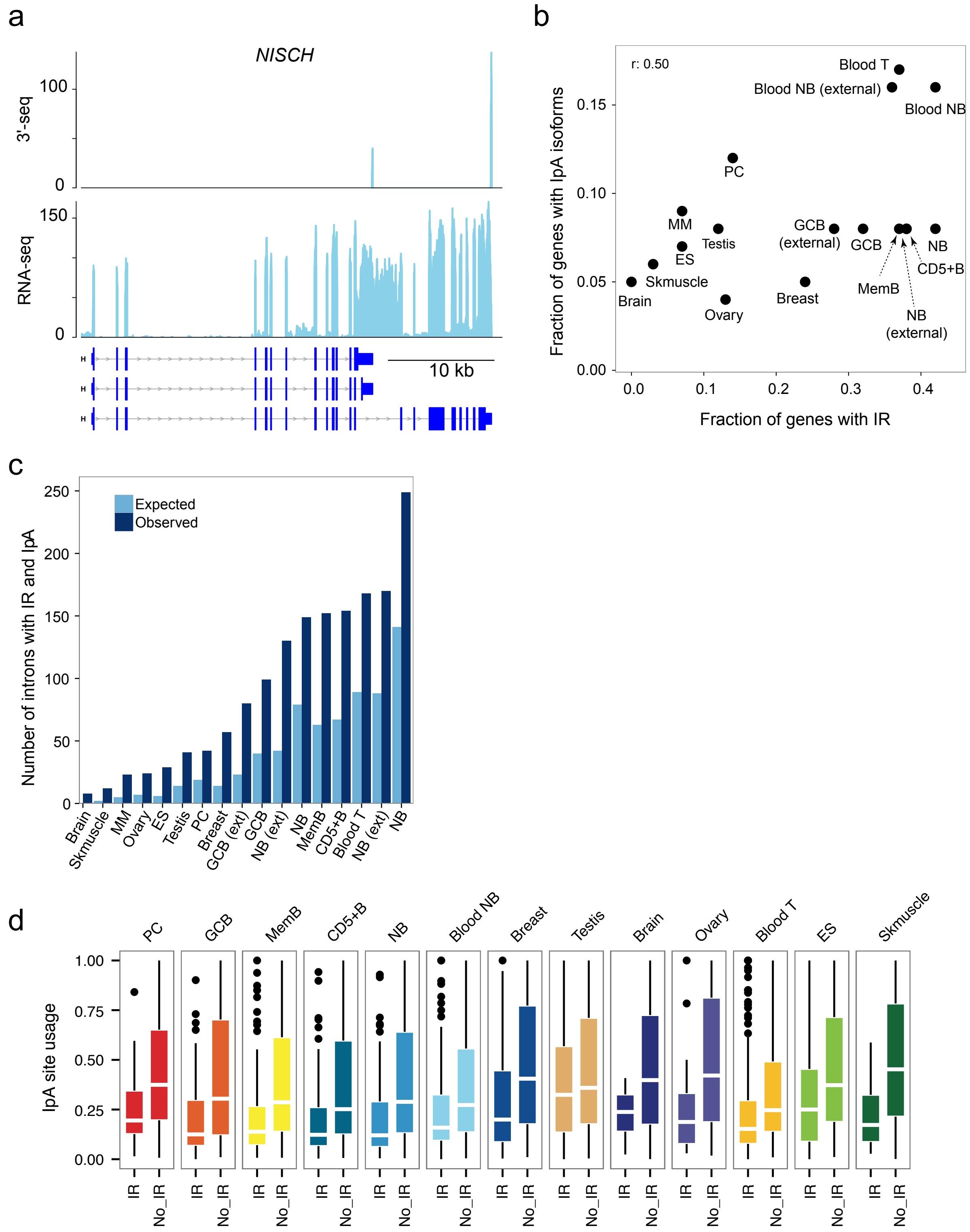

**Supplementary Figure 8: Co-occurrence of IpA events with intron retention.**

a) Co-occurrence of IpA with intron retention (IR). Shown as in Fig. 1a.

b) Correlation between IR and IpA isoform expression. Tissues with a higher number of genes with IR also have more genes that express IpA isoforms (Pearson correlation coefficient, r = 0.5).

b) Higher incidence of IpA events in introns with IR. The number of introns where IpA and IR are observed simultaneously is much higher than expected by chance.

c) IpA site usage differs between introns with or without IR. The distribution of IpA site usage was calculated for the two groups and plotted for each cell type. The median usage of the IpA isoforms that co-occurred with IR is lower compared to those occurring in introns that are not retained.

**Supplementary Table 1.** Characterization of normal human immune cells investigated by 3'-seq

| Sample | Derived from | Sample name | Markers for sorting | No of samples | Accession number |
|---|---|---|---|---|---|
| CD5+B | Tonsil | CD5+B3-CD5+B6 | CD5+, CD19+ | 4 | GSE111310 |
| NB | Tonsil | NB3-NB4 | CD19+, CD27- | 2 | GSE111310 |
| NB | Blood | NB1-NB2 | CD19+, CD27- | 2 | SRP029953 |
| MemB | Tonsil | M1-M2 | CD19+, CD27+ | 2 | GSE111310 |
| GCB | Tonsil | GC1-GC2 | CD19+, CD38+ | 2 | GSE111310 |
| PC | BM | PC1-PC2 | CD138+ | 2 | GSE111310 |
| T | Blood | T2-T3 | CD3+ | 2 | GSE111310 |

BM, bone marrow

**Supplementary Table 2.** MM patient characteristics

| | MM group | ISS stage at sample collection | Type | Cyto-genetics | RNA-seq | 3'-seq |
|---|---|---|---|---|---|---|
| MM1 | 3 | I | IgA | t(11;14) --> IGH-CCND1 | Y | Y |
| MM2 | 3 | I | IgA | t(11;14) --> IGH-CCND1 | Y | Y |
| MM3 | 1 | I | IgG LLC | t(4;14), loss of chr 13 and 17 and other complex cytogenetic changes | Y | Y |
| MM4 | 1 | III | IgG KLC | Hyperdiploid | Y | Y |
| MM5 | 2 | II | IgA KLC | t(11;14), del(13q), deletion P53 | Y | Y |
| MM6 | 1 | II | IgA KLC | t(11;14) --> IGH-CCND1 | Y | Y |
| MM7 | 2 | I | Kappa LC | t(11;14) --> IGH-CCND1 | Y | Y |
| MM8 | 1 | I | Kappa LC | t(11;14) --> IGH-CCND1 | Y | Y |
| MM9 | 1 | I | IgG | Hyperdiploid | Y | Y |
| MM10 | 2 | II | IgA KLC | t(11;14), del(13q), deletion P53 | Y | Y |
| MM11 | 1 | II | IgM KLC | t(11;14), del(1q), inv(9)(p12q13) | Y | Y |
| MM12 | 3 | II | IgM KLC | t(11;14), del(1q) | Y | Y |
| MM13 | 2 | II | IgG Lambda | Normal | N | Y |
| MM14 | 1 | I | IgG kappa | t(14;16) --> IGH-MAF | N | Y |
| MM15 | 3 | III | Non-secretory PC leukemia | Complex | N | Y |

N, No; Y, Yes

**Supplementary Table 3.** Characterization of normal human immune cells investigated by RNA-seq

| Sample | Derived from | Sample name | Markers for sorting | No of samples | Accession number |
|---|---|---|---|---|---|
| CD5+B | Tonsil | CD5+B3-CD5+B4 | CD5+, CD19+ | 2 | GSE111310 |
| CD5+B | Blood | CD5+B2 | CD5+, CD19+ | 1 | GSE111310 |
| NB | Tonsil | NB3-NB5 | CD19+, CD27- | 3 | GSE111310 |
| NB | Blood | NB1-NB2, NB6 | CD19+, CD27- | 3 | GSE111310 |
| MemB | Tonsil | M2, M6 | CD19+, CD27+ | 2 | GSE111310 |
| MemB | Blood | M3-M5 | CD19+, CD27+ | 3 | GSE111310 |
| GCB | Tonsil | GC1-4 | CD19+, CD38+ | 4 | GSE111310 |
| PC | BM | PC4-PC21 | CD138+ | 18 | GSE111310 |
| NB | Tonsil | | IgD+ | 4 | GSE45982 |
| GCB | Tonsil | | CD77+ | 4 | GSE45982 |
| NB | Blood | | CD19+ CD5- CD27- | 2 | ERX397853<br>ERX397892 |
| T | Blood | | CD3+ | | GSM1576415 |

Other 3'-seq profiles were from Lianoglou et al. 2013 (SRP029953).
