# Supplementary material for: Widespread intronic polyadenylation diversifies immune cell transcriptomes
Source: Nat Commun. 2018 Apr 30;9:1716. doi: 10.1038/s41467-018-04112-z (PMC5928244; doi:10.1038/s41467-018-04112-z)
Supplement: Supplementary file 3 — Description of Additional Supplementary Files [file 41467_2018_4112_MOESM3_ESM.pdf]

Description of Additional Supplementary Files

File Name: Supplementary Data 1
Description: IpA isoforms with significantly different usage of IpA site between NB cells vs other immune cell types and MM vs PC.
